# Novel concepts in virally induced asthma

**DOI:** 10.1186/1476-7961-7-2

**Published:** 2009-01-20

**Authors:** Matthew M Huckabee, R Stokes Peebles

**Affiliations:** 1 Division of Allergy, Pulmonary, and Critical Care Medicine, Department of Medicine, Vanderbilt University School of Medicine, Nashville, TN 37232-2650, USA

## Abstract

Viruses are the predominant infectious cause of asthma exacerbations in the developed world. In addition, recent evidence strongly suggests that viral infections may also have a causal role in the development of childhood asthma. In this article, we will briefly describe the general perception of how the link between infections and asthma has changed over the last century, and then focus on very recent developments that have provided new insights into the contribution of viruses to asthma pathogenesis. Highlighted areas include the contribution of severe early life viral infections to asthma inception, genetic determinants of severe viral infections in infancy, the differences in innate and adaptive immune system cytokine responses to viral infection between asthmatic and nonasthmatic subjects, and a potential vaccine strategy to prevent severe early life virally-induced illness.

## Background

Infections have been recognized to be a cause of asthma exacerbations for over 100 years. In his landmark textbook that heralded modern medicine, Sir William Osler noted that among other asthma exacerbating factors such as allergens and environmental changes, "every fresh cold" could induce a paroxysm of disease [1]. During Osler's time, viruses had not yet been isolated as infectious agents, and in the first half of the twentieth century, the "colds" or upper respiratory tract infections that caused worsening of asthma symptoms were largely presumed to be caused by a hypersensitivity or allergy to the bacteria that were considered to be responsible for these infections [2]. This concept was accepted by some physicians who attempted to desensitize patients who experienced asthma exacerbations following respiratory tract infections by administering allergy shots that contained bacterial extracts [3]. Detractors of the bacterial allergy theory pointed out that while extracts of environmental allergens such as pollens or animal dander could produce positive immediate skin test results in sensitive subjects, bacterial extracts did not provoke such hypersensitivity reactions [4].

Despite the inability of bacterial skin testing to confirm hypersensitivity to these organisms, immunotherapy containing bacterial extracts was used substantially in the prophylaxis against asthma exacerbations from the 1920s to the late 1950s [4,5]. Physicians who used this bacterial immunotherapy strategy reported that the majority of their patients had symptomatic improvement on this treatment [3,5]. However, several well controlled trials in the late 1950s and early 1960s revealed that immunotherapy to bacterial antigens was no more effective than placebo, and there was a call to end this practice that was widely heeded by the medical community [5-7]. A further blow to the concept of bacterial allergy came with randomized controlled-trials in the 1970s and 1980s which showed that antibiotic administration did not alter the course of asthma exacerbations, suggesting that bacteria were not pathogenic [8,9]. The bacterial allergy theory presents an interesting irony in the history of immunotherapy when viewed in light of the hygiene hypothesis. The hygiene hypothesis suggests that an early exposure to bacterial products (i.e. endotoxin) may prevent subsequent allergic sensitization and asthma because the immune system has been steered toward the Th1 pathway of CD4 development and away from a Th2 phenotype. An interesting test of the hygiene hypothesis would have been to have given bacterial allergen immunotherapy containing endotoxin to young children, rather than adults as occurred in the randomized trials that proved this form of immunotherapy was not efficacious in the studied older population. The hygiene hypothesis would predict that bacterial allergen immunotherapy might have reduced allergic disease and asthma in children that received this treatment.

To further determine if the presence of bacteria was associated with worsening asthma symptoms, investigators sought to identify if bacteria could be cultured from the upper respiratory tract more often when patients were symptomatic with asthma exacerbations, compared to times when they were not. There was no difference in the presence of bacterial infections when subjects were symptomatic compared to when they were not, further suggesting that bacteria were not responsible for asthma worsening [10,11]. In contrast, studies using techniques that are relatively insensitive for determining the presence of viral infection of the upper respiratory tract, such as culture, immunofluorescence, and serology, revealed that viruses were present from 2 to 5 times more frequently when asthmatic subjects were in exacerbation compared to when they were without symptoms [12-15].

By the mid 1990s, polymerase chain reaction (PCR) technology was being used in clinical research studies and revealed that viruses could be detected in the respiratory tract secretions of 80–85% of school children who were experiencing asthma exacerbations [16]. Studies of adult asthmatics also revealed that viruses could be detected in subjects' respiratory tract secretions during 50–80% of exacerbations [17]. In both school aged children and adults, rhinovirus (RV) is the most common virus detected during exacerbations [18,19]. Thus, we have come full circle in that in the 1890s Osler recognized the cold to be a major cause of asthma exacerbations and we have now recently recognized that the etiologic agent most often responsible for colds, rhinovirus, is also the most frequent cause of asthma exacerbations. Now that the historical perspective linking viral infection and exacerbations has been explored, we will focus on selected papers published in the last two years which further elucidate the role and mechanisms of viral infections in asthma development and pathogenesis.

## Asthma inception

The role of viral infections in asthma inception in infants and young children has been debated for many years. Wu and colleagues found that birth timing in relationship to the peak of hospitalization for bronchiolitis determines the risk of severe bronchiolitis during infancy, as well as the risk of developing asthma [20]. These findings suggest that bronchiolitis, or some factor closely associated with bronchiolitis, might cause asthma. In this investigation, a cohort of over 95,000 children enrolled in the Tennessee Medicaid program who were born between 1995 and 2000 was followed over their first five viral seasons until the age of 5 1/2 years. This population represents approximately one-quarter of the births each year in that state. The authors conducted six analyses to determine how the effect of infant birth date in relationship to the winter virus peak might alter the risk of developing early childhood asthma.

**Figure 1:**
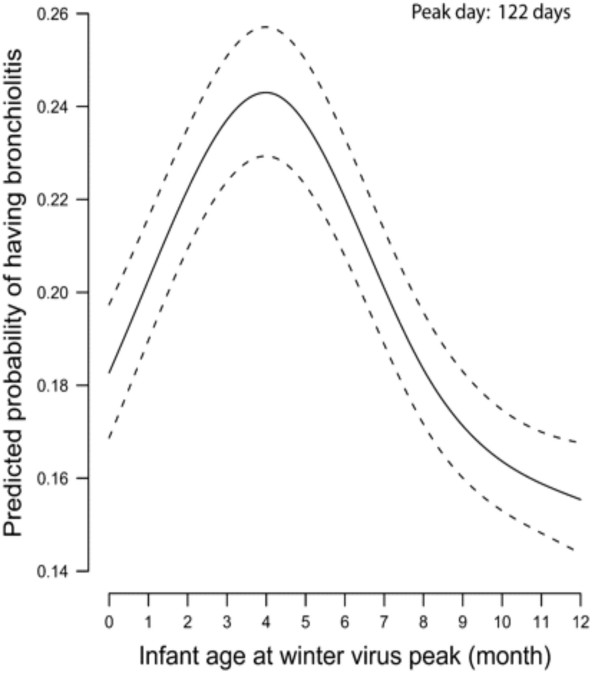
**Predicted probability and 95% confidence intervals of bronchiolitis requiring a health care visit during infancy (hospitalization, emergency department visit, or outpatient visit) by infant age in months at the winter virus peak ($\chi_{3}^{2}$ = 345.52; *P *< 0·001)**. Results were obtained from a multivariable logistic regression model. Effect was adjusted for gender, infant race, birth weight, gestational age, number of living siblings, region of residence, maternal smoking, marital status, maternal education, and season. Reprinted with permission from Wu et al, Am J Respir Crit Care Med 178:1123–1129, 2008.

**Figure 2:**
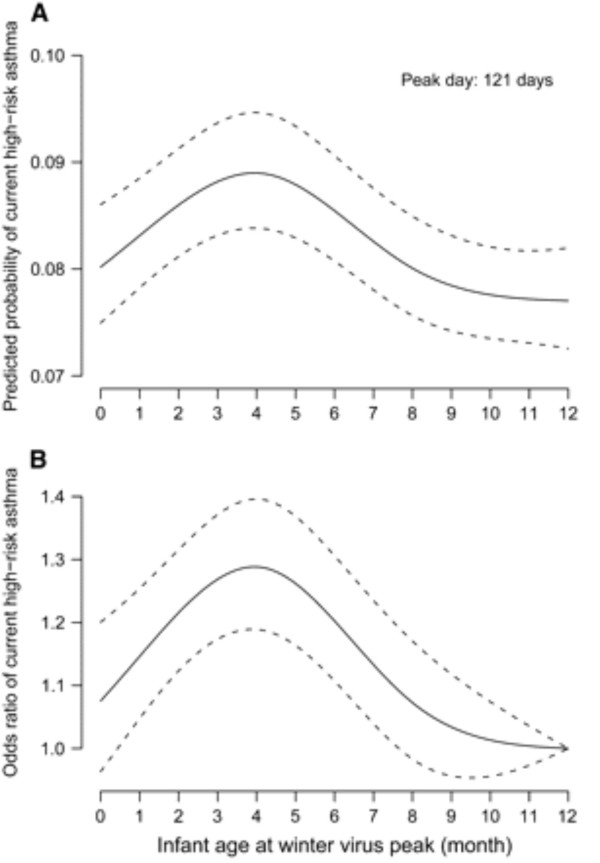
**Differential risk of developing current high-risk childhood asthma in relationship to infant age at the winter virus peak**. Results were obtained from a multivariable logistic regression model adjusted for gender, infant race, birth weight, gestational age, number of living siblings, region of residence, maternal smoking, marital status, maternal education, and season. (*A*) Predicted probability and 95% confidence intervals (CI) of developing current high-risk childhood asthma by infant age in months at the winter virus peak ($\chi_{3}^{2}$ = 49.05; *P *< 0·001). The area under the curve is equal to the asthma prevalence of the population. (*B*) Adjusted odds ratio and 95% CI of developing current high-risk childhood asthma relative to children who were 12 months of age at the winter virus peak. Infants who were 1 year of age at the winter virus peak served as the reference group. Reprinted with permission from Wu et al, Am J Respir Crit Care Med 178:1123–1129, 2008.

First, the timing of infant birth in relationship to the winter virus peak, as defined as the first day of the week with the highest number of bronchiolitis hospitalizations for that winter season, predicted the likelihood of developing clinically significant bronchiolitis [21]. Clinically significant bronchiolitis was defined as hospitalization, emergency department visit, or outpatient visit. After adjusting for other factors previously reported to be associated with severe bronchiolitis, infants 122 days (95% CI, 118–126 days) old at the winter virus peak had the greatest risk of developing clinically significant bronchiolitis (Figure 1). Second, the timing of infant birth in relationship to the winter virus peak predicted the likelihood of developing childhood asthma as defined by ICD-9 code or medication use for asthma. Children who were 121 days (95% CI, 108–131 days) old at the winter virus peak had the greatest risk of developing high risk asthma when a comparison was made with children who were either older or younger at the peak of the winter virus season (Figure 2) [22]. High risk asthma was defined as asthma-related hospitalization, emergency department treatment, or rescue corticosteroid prescription. Despite the fact that the winter virus peak shifted as much as six weeks over the 5 viral seasons studied for each child, this relationship of age at peak viral season with the subsequent development of asthma was not affected when subgroup analysis was conducted on children who encountered early or late winter virus peaks. Surprisingly, maternal history of asthma had no effect on this analysis.

In the third analysis, compared to children who were 365 days old at the winter virus peak, there was a 29% increase in odds of developing high-risk childhood asthma for children who were 121 days of age at the winter virus peak, the almost identical infant age at winter virus peak that conferred the greatest risk of bronchiolitis [23]. The importance of age at the time of virus peak was further strengthened by the fourth analysis which found that the age at which a child first presented with bronchiolitis symptoms had no bearing on the subsequent development of asthma; however, the age of the child in relationship to the peak of the viral season conferred an increased likelihood of developing high risk asthma.

Fifth, despite the fact that smoking during pregnancy and maternal asthma increased the odds of developing high risk asthma by 7% and 82%, respectively, neither of these factors significantly changed the relationship between infant age at winter virus peak and childhood asthma [24]. There were no interactions between infant age at the winter virus peak and other covariates examined, such as infant race, gender, birth weight, gestational age, other living siblings, region of residence, and maternal education on the risk of developing asthma [25].

The authors speculate that there are two possible explanations for the relationship between the timing of the peak of the viral season and the increased risk of developing both significant bronchiolitis and high risk asthma [26]. The first is that a common genetic predisposition could lead to both severe winter viral bronchiolitis and asthma, while the other is that an environmental exposure such as winter viral infection leads to asthma. The data presented does not rule out either or both of these possibilities. One of the most interesting aspects of this study is the risk of developing either bronchiolitis or asthma by timing of birth in relationship to the winter virus peak was present for every year analyzed, despite the fact that the timing of the viral peak shifted from year to year. Thus, being born around 4 months (120 days) before the peak of the viral season confers a similar and significantly increased risk of developing both bronchiolitis and subsequent asthma [27].

Wu and colleagues did not determine the viruses responsible for the peak of the viral season in their study [28]; however, other long-term longitudinal studies have reported that respiratory syncytial virus (RSV) lower respiratory tract infection increases the risk for subsequent wheezing [29] and asthma [30] at least through age 10. However, Jackson and colleagues have found that rhinovirus infections that result in wheezing illness in the first three years of life predict asthma development in children that are deemed high risk because of a family history of allergy or asthma, and that this risk surpasses that associated with early life wheezing from RSV infection [31]. In this study, 259 children enrolled in the University of Wisconsin Childhood Origins of Asthma (COAST) study were followed prospectively for 6 years and had met entry criteria because either one or both parents had a history of positive skin tests to aeroallergens or physician diagnosed asthma [32]. The presence of virus in respiratory tract secretions in early life was determined by PCR and standard viral detection techniques at scheduled monthly intervals during the first year of life and when subjects developed respiratory tract illnesses. The assays incorporated potential detection for rhinovirus, RSV, parainfluenza virus types 1–4, influenza types A and B, adenovirus, enterovirus, coronavirus, and metapneumovirus.

**Figure 3:**
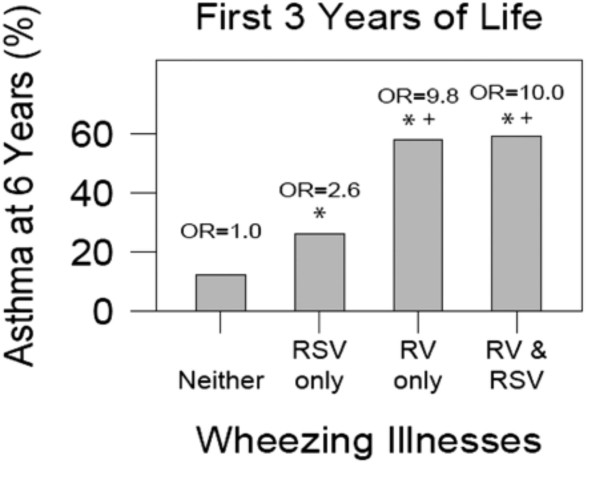
Risk of asthma at age 6 years in children who wheezed during the first 3 years of life with rhinovirus (RV), respiratory syncytial virus (RSV), or both (*P < 0.05 vs. Neither; +*P *< 0.05 vs. RSV only). OR = odds ratio. Reprinted with permission from Jackson et al, Am J Respir Crit Care Med 178:667–672, 2008.

Analysis of respiratory tract secretions revealed that viruses were present in these fluids 90% of the time that wheezing illnesses occurred in the first three years of life [33]. Rhinovirus was the virus most frequently detected (48%), followed by RSV (21%), parainfluenza viruses (12%), with the other viruses being detected in less than 10% of the respiratory tract secretions. In approximately 10% of wheezing illnesses, more than one virus was detected in the respiratory tract secretions. In this high risk population, 28% of the subjects met the criteria for asthma diagnosis at age 6 based on National Asthma Education and Prevention program guidelines. When an analysis was performed to determine which viruses were most often detected in the respiratory tract secretions during wheezing illnesses before age three and associated with the diagnosis of asthma at age 6, rhinovirus was the most common virus identified. The odds ratio of having asthma at age 6 was 9.8 for rhinovirus, and 2.6 for RSV, respectively, when these viruses were detected during wheezing illnesses that occurred at age 3 or before (Figure 3). The referent was neither RSV nor RV being detected in the nasal secretions. When both rhinovirus and RSV were detected, the odds ratio of having asthma at age 6 was 10.0. Wheezing illness with none of the other viruses included in the viral detection assays was associated with asthma diagnosis at age 6.

The authors also analyzed the data based on the viruses detected at each year up to age 3 and the subsequent risk of developing asthma at age 6 [34]. They found that rhinovirus detection in the respiratory tract secretions during wheezing illness during the first year of life was associated with a statistically significant increased odds ratio (2.7) for having an asthma diagnosis at age 6, and this was comparable to the odds ratio (3.6) of having asthma at age 6 that was associated with skin test positivity to aeroallergens. There was a further increase in the odds ratio of having asthma at age 6 associated with rhinovirus in the respiratory tract secretions during wheezing illnesses at age 2 (OR 6.5) and age 3 (31.7). In contrast, there was no increase in the odds ratio of having asthma at age 6 with RSV detected during wheezing illnesses at either age 1 or age 2, and an increased odds ratio with RSV detection was only seen in the third year of life (9.9). One could make the strong argument that while wheezing at age 1, and perhaps at age 2, may most likely represent bronchiolitis, virally-induced wheezing at age 3 may more likely reflect an exacerbation of asthma. Of note, subjects with acute RV infection associated with wheezing from age 1–3 years had a 59% asthma risk at age 6 [35]. In any case, this study proposes that rhinovirus infection in early life may be causative for the subsequent development of asthma and that future studies examining the relationship between infant infections and asthma needs to include assays for the detection of rhinovirus and the inclusion of this virus in the data analysis [36].

## Genetic susceptibility to bronchiolitis

As noted above, early life rhinovirus and RSV are associated with the subsequent development of asthma. The recognition that rhinovirus may have an important role in the inception of asthma is a novel concept, first put forward by Jackson and colleagues as just reviewed [37]. Therefore genetic association studies examining the host risk factors for severe rhinovirus infection in early life have not yet been performed. However, there has been a great deal of interest in genetic determinants of severe RSV-induced disease, mainly because of the concept that the host immune response is a large determinant of severity of illness, although as we will mention later viral determinants may also have an important contribution to disease severity. Over the last several years, genetic association studies suggest that variation in specific genetic loci confer susceptibility to RSV-induced illness. Host response genes that have been associated with RSV disease include surfactant protein A, surfactant protein D, toll like receptor (TLR) 4, tumor necrosis factor (TNF), interleukin (IL)-4, IL-9, IL-10, IL-8, IL-13, and RANTES [38-45]. Recently, Janssen and colleagues performed a large scale genotyping study in the Netherlands using a candidate gene approach to identify potentially new genes and biologic pathways that contribute to susceptibility to severe RSV infection [46]. They reported that genetic susceptibility to RSV is predominantly associated with innate immune genes. In their analysis, these investigators performed a genetic association study in 470 children who had been hospitalized for RSV bronchiolitis. In order to rule our potential confounding factors, children with a history of airway morbidity, airway medication, and wheeze were excluded from the study. In the analysis, the enrolled children's parents and 1008 random, population controls were also included.

Janssen reported that 22 single nucleotide polymorphisms (SNPs) in 21 genes were identified that were statistically significantly associated with severe RSV disease at either the genotype or allele level [47]. Those SNPs associated with severe RSV disease both at the genotype and allele level included the genes for the vitamin D receptor (*VDR*), the signal transduction molecule Jun (*JUN*) which composes part of the AP-1 signaling complex, interferon (IFN)-α (*IFNA5*) which is a type I IFN involved in early cytokine responses to viral infection, inducible nitric oxide synthase (*NOS2A*), and the high affinity IgE receptor (*FCER1A*) important in IgE binding to mast cells, basophils, dendritic cells, and other immune cells.

SNPs were also discovered that were associated with severe RSV disease at the allele level [48]. Based on the function of the gene products, the investigators divided the SNPs into four subgroups: innate immunity (*IFNA13*, *IL15*, *STAT1*, and *TLR8*), chemotaxis (*CCL8*, *ITGB2*, and *VCAM*), adaptive immunity (*CD28 *and *STAT1*), and allergic asthma (*MS3A2*, *ADAM33*, *IL4R*, and *IL9R*). Those SNPs that had an association with RSV at the genotype level only were present in genes involved in innate immunity (*TNF *and *NCF2*) and adaptive immunity (*IL10*, *IL4R*, and *IL17*).

This study is an important contribution to our scientific knowledge of RSV-induced illness because it provides candidates for future genetic analysis, haplotype determination, and functional studies to further define the contributing factors that are responsible for severe disease. In addition, the data reveal that genetic susceptibility to RSV bronchiolitis is a very complex trait with host response genes being strong contribution factors.

## Cytokine responses to viral infection

As just mentioned above, genes associated with severe RSV disease in infancy, and perhaps asthma inception, include those that are involved in cytokine production (*IFNA13*, *IL15*, *IL10*, and *IL17*) and cytokine receptor signaling (*IL4R*,*IL9R*, and *STAT1*). Since there is a very high percentage of asthmatic children who are atopic, one could hypothesize that those children who have RSV bronchiolitis in infancy and who subsequently develop asthma have increased levels of Th2 cytokines compared to children who experience RSV bronchiolitis, but that are not diagnosed with asthma. Castro and colleagues tested this hypothesis in a prospective cohort of 206 previously healthy children who had been enrolled in the RSV Bronchiolitis in Early Life (RBEL) study [49]. As opposed to the COAST study mentioned earlier [50], these children were not preselected based on a family history of allergy or asthma [51]. Despite this, there was an extraordinary high percentage (48%) of children enrolled in RBEL who were physician diagnosed with asthma at age 6. Thirty-two percent of children had positive skin tests at age 3; however, there was not an RSV-uninfected group that could serve as controls to determine how the allergic sensitization rate varied based on early infant severe RSV infection. Contrary to the investigators' hypothesis, there was no difference in cytokine generation between those children who had severe RSV bronchiolitis and who developed asthma and those who were not given an asthma diagnosis. At age 6 years, asthmatic children who had experienced severe RSV infection during infancy actually had decreased IL-13 levels (defined by peripheral blood T cells that were stimulated with phorbol and ionomycin) compared to nonasthmatic subjects. In addition, there were no differences in IFN-γ, IL-2, or IL-4 levels from stimulated T cells between the asthmatic and nonasthmatic groups [52]. However, other studies suggest that cytokine responses occurring during bronchiolitis do predict subsequent asthma development. For instance, Renzi and colleagues found that infants with later possible and probable asthma had decreased peripheral blood mononuclear cell production of IFN-γ during bronchiolitis [53].

The cytokine profile produced in bronchiolitis is very different from that which occurs with virally-induced asthma exacerbations. Message and colleagues performed a study in which they experimentally infected 10 asthmatic subjects and 15 nonasthmatic subjects with rhinovirus and then examined pulmonary function and cytokine responses in CD4 cells from peripheral blood and bronchoalveolar lavage fluid [54]. They report that asthmatic subjects had significantly increased chest symptoms, a greater fall in the forced expiratory volume in 1 second (FEV_1_) and peak expiratory flow (PEF), and heightened methacholine reactivity in response to experimental rhinovirus infection compared to the nonasthmatic subjects. In regard to cytokine responses, persons who had greater IFN-γ and IL-10 production by peripheral blood CD4 cells had decreased viral loads and decreased upper respiratory tract symptoms. Similarly, those subjects with greater IFN-γ production by CD4 cells obtained by BAL were protected from falls in PEF after rhinovirus infection, while those subjects whose BAL CD4 cells produced higher levels of the Th2 cytokines IL-4, IL-5, and IL-13 all had increased lower respiratory symptom scores. In addition, after rhinovirus infection, BAL cells from asthmatic subjects were deficient in the production of IFN-γ, IL-10, and IL-12, while producing greater Th2 cytokine levels. In contrast rhinovirus infection resulted in induced IFN-γ, IL-10, and IL-12 from the nonasthmatics' BAL CD4 cells, while there was no increase in Th2 cytokines in this group [55]. This data fits very well with prior reports from this group which suggests that there are significant defects in the cytokine responses of the innate immune system in asthmatic subjects compared to nonasthmatics. These investigators previously showed that the airway epithelium from asthmatics produced significantly decreased amounts of type I [56] and type III interferons [57] in response to rhinovirus infection compared to the epithelial cells from nonasthmatics. This same group has also recently reported that rhinovirus infection of epithelial cells results in a dramatic increase in pro-inflammatory cytokine production with near concurrent exposure to the major allergen from the dust mite species Dermatophagoides pteronyssinus [58]. In this study, the immortalized line of human bronchial epithelial cells, BEAS-2B, were exposed to rhinovirus, dust mite antigen, or both. When rhinovirus infection occurred 24 hours prior to dust mite exposure, there was a synergistic increase in epithelial cell production of IL-8, an important neutrophil chemotactic and survival factor. This rhinovirus/dust mite exposure pattern was associated with early synergistic NF-kB translocation induction, perhaps explaining the augmented IL-8 release. There was also increased expression of the adhesion molecule ICAM-1, which serves as the major rhinovirus receptor on epithelial cells. This synergism in IL-8 expression and NF-kB translocation was not observed when epithelial cell dust mite exposure occurred prior to rhinovirus infection, suggesting that the timing of these exposures is critical to cytokine elaboration [59].

## Therapy

As early life severe viral infection has been linked to asthma inception, preventing such episodes would perhaps decrease the incidence and prevalence of asthma. Unfortunately there is no currently available FDA-approved vaccine against RSV. However, a recently completed trial provides hope that such a vaccine approach may soon be available. Wright and colleagues report that 388 study subjects were examined and divided into one of three groups: children that received one of several live, attenuated RSV vaccines, children administered placebo vaccine, and age-matched controls [60]. Subjects that were vaccinated with the live, attenuated RSV vaccines had a significant decrease in RSV-associated upper respiratory tract illness compared to those that were administered a placebo or nonvaccinated subjects. Results stratified for subject age revealed that RSV-associated upper respiratory tract illness occurred in 6–24 month old children with a frequency of 14% in those receiving the RSV vaccine and in 20% of the controls. When infants were examined, 16% of those vaccinated with the RSV vaccine had RSV-associated upper respiratory tract illness compared to 25% in the control groups. While these results are promising, there are some caveats that need to be considered. First, the study was not randomized, nor was it blinded. Second, the dates of surveillance ranged over the period from 1994–2003 and incorporated seven different live, attenuated vaccines. However, it is important to note that the RSV vaccines did not result in the enhanced illness after naturally occurring infection that was seen in children who had previously received an alum-precipitated, formalin-inactivated vaccine in the late 1960s [61].

While, live attenuated RSV vaccines may present a potential novel therapy, other studies suggest that prophylactic anti-RSV immunoglobulin may affect the later development of asthma. Simoes reported that palivizumab, an anti-RSV monocolonal antibody, reduced subsequent wheezing in preterm infants [62]. In a retrospective analysis, Wenzel and colleagues found that children at risk for RSV bronchiolitis that were treated with RSV-immune globulin had a decreased incidence of obstructive airway disease, ever having an asthma exacerbation, and atopic disease as defined by skin testing to aeroallergens, compared to a control group that did not receive RSV immunotherapy [63]. To fully understand the effect of anti-RSV immunoflobulin on the later development of allergic disease and asthma, a prospective, randomized, double-blinded, placebo-controlled trial needs to be conducted.

## Summary

Although bacterial infections were long considered to the primary infectious agents linked to asthma pathogenesis, over the last decade, new data supports that virus infections are the cause of a great majority of asthma exacerbations. New data published in the last year strengthens earlier reports that early life viral infections may have a significant role in asthma inception. Gene association studies reveal that persons with deficiencies in innate immunity may be more predisposed to these severe early life episodes of viral bronchiolitis that predispose to the later development of asthma. Deficiencies in innate immunity are also present in adult asthmatics compared to their nonasthmatic counterpart as witnessed by the difference in cytokine responses in peripheral blood and BAL CD4+ cells following rhinovirus infection and in the epithelial cells responses to viral challenge. Prevention of early life severe bronchiolitis episodes is a major priority and vaccine approaches are being developed to fill this therapeutic need. Further research and therapeutic strategies are needed to advance our understanding of the contribution of viruses to asthma pathogenesis and protect against both the inception and exacerbations of this disease.

## Abbreviations

PCR: polymerase chain reaction; RSV: respiratory syncytial virus; TLR: toll like receptor; TNF: tumor necrosis factor; IL: interleukin; SNP: single nucleotide polymorphism; VDR: vitamin D receptor; IFN: interferon, NOS2A: nitric oxide synthase; FEV1: forced expiratory volume in 1 second; PEF: peak expiratory flow; RV: rhinovirus.

## Competing interests

The authors declare that they have no competing interests.

## Authors' contributions

MMH and RSP both wrote the paper and contributed equally.
